# Address of the President Texas State Medical Association in Interest of the International Medical Congress

**Published:** 1887-06

**Authors:** Sam. R. Burroughs

**Affiliations:** President Texas State Medical Association; Executive Office Raymond, Texas


### TEXAS STATE MEDICAL ASSOCIATION.

[Published by request.]

EXECUTIVE OFFICE RAYMOND, TEXAS, May 18. 1887.

*To Members of the Texas State Medical Association,*

GENTLEMEN:—I am in receipt of a communication from Dr. R. J. Dunglison, of Philadelphia, Chairman of Finance Committee, Ninth International Medical Congress. The following excerpt from the circular letter will speak for itself:

"As Chairman of the Finance Committee of the Ninth International Medical Congress, I desire to invite your attention to the importance of the members of your Society, either individually or collectively, sustaining the credit of the Medical Profession and the Government of the United States, by contributing to the funds of the Congress, to enable its officers to meet its expenses in a manner becoming this great Nation, and to reciprocate the courtesies so liberally bestowed upon delegates from the United States to previous meetings of the Congress in Europe."

It is needless to say that much regret is entertained because of the late hour in which this intelligence reaches us, since its claims are debarred the privilege and advantage of being presented to the Association in Convention assembled.

It, therefore, becomes my pleasant duty to present to the individual membess of our corporate body, the merits of its important communication; and, in doing so, I feel assured that no elucidation on my part is necessary to a clear and full comprehension of the specific environment engendered by this legitimate and worthy appeal.

That the approaching session of the Ninth International Medical Congress should be made a grand success in all its appointments, no one wiil deny. That the Medical Profession of Texas should contribute to this success whenever and wherever the demand may be declared, is also an important truth. That whatever action is taken in the premises, on the part of the members, must be executed at an early date in order that it may be effective in its purpose, is an additional truth.

Whatever reflects upon our National Government will be felt in Texas. Whatever affects the Profession of the United States touches the Profession of Texas. In matters both Medical and International, Texas is interested.

In view of the above facts, I would respectfully recommend that each individual member address his financial ablity, his professional, state and national pride, his devotion to the cause of Science and make, accordingly the proper assessment, and forward the same immediately to Dr. J. Larendon, Treasurer, Texas State Medical Association, Houston, Texas, who is hereby requested and authorized to remit the aggregate. to be determined, not later than the 30th of June, to Dr. R. J. Dunglison, Chairman Finance

Committee, Ninth International Medical Congress, 814 North Sixteenth Street, Philadelphia, Pa.

It is confidently expected that the members will not permit the escutcheon of Texas Medicine to be shadowed by a neglect of duty.

Your obedient servant,

SAM. R. BURROUGHS, M. D.
President Texas State Medical Association.

